# Histamine H_3_ Heteroreceptors Suppress Glutamatergic and GABAergic Synaptic Transmission in the Rat Insular Cortex

**DOI:** 10.3389/fncir.2017.00085

**Published:** 2017-11-09

**Authors:** Hiroki Takei, Kiyofumi Yamamoto, Yong-Chul Bae, Tetsuo Shirakawa, Masayuki Kobayashi

**Affiliations:** ^1^Department of Pharmacology, Nihon University School of Dentistry, Chiyoda-ku, Japan; ^2^Department of Pediatric Dentistry, Nihon University School of Dentistry, Chiyoda-ku, Japan; ^3^Division of Oral and Craniomaxillofacial Research, Dental Research Center, Nihon University School of Dentistry, Chiyoda-ku, Japan; ^4^Department of Oral Anatomy and Neurobiology, School of Dentistry, Kyungpook National University, Daegu, South Korea; ^5^Molecular Dynamics Imaging Unit, RIKEN Center for Life Science Technologies, Kobe, Japan

**Keywords:** histamine, heteroreceptor, H_3_, insular cortex, paired whole-cell recording

## Abstract

Histamine H_3_ receptors are autoreceptors that regulate histamine release from histaminergic neuronal terminals. The cerebral cortex, including the insular cortex (IC), expresses abundant H_3_ receptors; however, the functions and mechanisms of H_3_ receptors remain unknown. The aim of this study was to elucidate the functional roles of H_3_ in synaptic transmission in layer V of the rat IC. Unitary excitatory and inhibitory postsynaptic currents (uEPSCs and uIPSCs) were obtained through paired whole-cell patch-clamp recording in cerebrocortical slice preparations. The H_3_ receptor agonist, R-α-methylhistamine (RAMH), reduced the uEPSC amplitude obtained from pyramidal cell to pyramidal cell or GABAergic interneuron connections. Similarly, RAMH reduced the uIPSC amplitude in GABAergic interneuron to pyramidal cell connections. RAMH-induced decreases in both the uEPSC and uIPSC amplitudes were accompanied by increases in the failure rate and paired-pulse ratio. JNJ 5207852 dihydrochloride or thioperamide, H_3_ receptor antagonists, inhibited RAMH-induced suppression of uEPSCs and uIPSCs. Unexpectedly, thioperamide alone increased the uIPSC amplitude, suggesting that thioperamide was likely to act as an inverse agonist. Miniature EPSC or IPSC recordings support the hypothesis that the activation of H_3_ receptors suppresses the release of glutamate and GABA from presynaptic terminals. The colocalization of H_3_ receptors and glutamate decarboxylase or vesicular glutamate transport protein 1 in presynaptic axon terminals was confirmed through double pre-embedding microscopy, using a combination of pre-embedding immunogold and immunoperoxidase techniques. The suppressive regulation of H_3_ heteroreceptors on synaptic transmission might mediate the regulation of sensory information processes, such as gustation and visceral sensation, in the IC.

## Introduction

The histaminergic system plays a critical role in physiological brain functions, such as the regulation of sleep and wakefulness, thermogenesis, and learning and memory (Haas et al., 2008; Panula and Nuutinen, 2013). The tuberomammillary nucleus (TMN) in the hypothalamus is the source of histaminergic neurons (Panula et al., 1984; Watanabe et al., 1984), which project to nearly the entire central nervous system (Köhler et al., 1985; Haas and Panula, 2003). Histaminergic neurons in the TMN abundantly express histamine H_3_ receptors on the somata, dendrites, and axons (Haas et al., 2008). H_3_ receptors are coupled to the G_i/o_ protein (Clark and Hill, 1996), which reduces cAMP concentrations and modulates Ca^2+^ conductance (Takeshita et al., 1998). As autoreceptors, TMN H_3_ receptors critically regulate neural firing and histamine synthesis and release (Arrang et al., 1983, 1985). Several studies have reported another function of H_3_ receptors as presynaptic heteroreceptors, which modulate the release of other neurotransmitters, i.e., acetylcholine, noradrenaline, glutamate, and GABA (Brown et al., 2001). The dysfunction of H_3_ receptors causes various neurological disorders, including movement disorders, hyperphagia, neuroinflammation, and sleep disorders (Haas et al., 2008; Panula and Nuutinen, 2013). Therefore, H_3_ receptors have recently received attention as potential targets for drug development against neurological disorders.

Histamine modulates higher brain functions processed in the cerebral cortex (Haas et al., 2008; Panula and Nuutinen, 2013). Among the histaminergic receptor subtypes, i.e., H_1_, H_2_, and H_3_, the functions of H_2_ receptors have been well explored in the cerebral cortex. Intracellular recordings using slice preparations have demonstrated that H_2_ receptors block Ca^2+^-dependent K^+^ channels that regulate afterhyperpolarization (AHP) (McCormick and Williamson, 1989; McCormick et al., 1993), thereby increasing the frequency of repetitive spike firing (Takei et al., 2012). In contrast, little information is available about the functional roles of cortical H_3_ receptors, although studies using autoradiography with [^125^I]iodoproxyfan and H_3_ receptor mRNA expression have demonstrated the high-density expression of H_3_ receptors in the cerebral cortex (Pillot et al., 2002). In the insular cortex (IC), which plays an important role in gustatory and visceral information processing (Yamamoto, 1984), the injection of an H_3_ receptor agonist, R-α-methylhistamine (RAMH), impairs aversive taste memory formation (Purón-Sierra et al., 2010). This physiological result corroborates the anatomical findings that the IC receives histaminergic projections from the TMN (Haas et al., 2008), and expresses abundant H_3_ receptors throughout all layers (Pollard et al., 1993; Cumming et al., 1994; Pillot et al., 2002). Considering that H_3_ receptors regulate excitatory synaptic transmission in the hippocampus (Brown and Haas, 1999), basolateral amygdala (Jiang et al., 2005), and striatum (Doreulee et al., 2001), it is likely that the H_3_ receptor-mediated modulation of EPSPs plays a critical role in neuroplasticity in the cerebral cortex. In addition, GABAergic synaptic transmission, which regulates ocular dominance plasticity in the adolescent visual cortex (Hensch et al., 1998), might contribute to the induction of these changes. It is worth noting that H_3_ receptor expression labeled with [^3^H](*R*)α-methylhistamine increases from postnatal days 9–16, and reaches the adult level on postnatal days 23 in the cerebral cortex (Ryu et al., 1995). This developmental profile of H_3_ receptor expression is contradict to that of histamine concentration showing very high content at birth and a steep decrease to the adult level by postnatal days 16. However, almost no information is available on the H_3_ receptor-dependent modulation of synaptic transmission in the developing cerebral cortex.

The present study aimed to investigate H_3_ receptor-mediated histaminergic modulatory mechanisms of excitatory and inhibitory local circuits comprising pyramidal and GABAergic cells in the developing IC using paired whole-cell patch-clamp recordings. The localization of H_3_ receptors in the glutamatergic and GABAergic terminals was examined through electron microscopy and immunohistochemistry to examine the location of H_3_ receptors in the IC. Furthermore, to elucidate the physiological function of H_3_ receptors, the effects of an H_3_ receptor agonist on excitatory propagation were examined using an *in vivo* optical imaging technique.

## Materials and Methods

All experiments were performed in accordance with the National Institutes of Health Guidelines for the Care and Use of Laboratory Animals, and the procedures were approved by the Institutional Animal Care and Use Committee of Nihon University. All efforts were made to minimize the number of animals used and their suffering.

### Slice Preparation

The techniques for preparing and maintaining rat cortical slices *in vitro* were similar to previously described methods (Koyanagi et al., 2010; Yamamoto et al., 2010; Kobayashi et al., 2012). Briefly, vesicular GABA transporter (VGAT)-Venus line A transgenic rats (Uematsu et al., 2008) of either sex, aged from 16 to 32 days old, were deeply anesthetized using sevoflurane (5%, Pfizer, Tokyo, Japan) and decapitated. The tissue blocks, including the IC, were rapidly removed and stored for 3 min in ice-cold modified artificial cerebrospinal fluid (ACSF) composed of the following (in mM): 230 sucrose, 2.5 KCl, 10 MgSO_4_, 1.25 NaH_2_PO_4_, 26 NaHCO_3_, 0.5 CaCl_2_, and 10 D-glucose. The coronal slice preparations (350 μm thickness) were obtained from the IC 0–1050 μm caudally from the middle cerebral artery using a microslicer (Linearslicer Pro 7, Dosaka EM, Kyoto, Japan) and incubated at 32°C for 40 min in a submersion-type holding chamber containing 50% modified ACSF and 50% normal ACSF (pH 7.35–7.40). Normal ACSF contained the following (in mM): 126 NaCl, 3 KCl, 2 MgSO_4_, 1.25 NaH_2_PO_4_, 26 NaHCO_3_, 2 CaCl_2_, and 10 D-glucose. Modified and normal ACSF were continuously aerated with 95% O_2_/5% CO_2_. The slices were subsequently placed in normal ACSF at 32°C for 1 h and maintained at room temperature until further use for recording.

### Cell Identification

The slices were transferred to a recording chamber continuously perfused with normal ACSF at a rate of 2.0–2.5 ml/min. Dual or triple whole-cell patch-clamp recordings were obtained from Venus-positive fluorescent neurons and pyramidal cells identified in layer V of the granular or dysgranular IC using a fluorescence microscope equipped with Nomarski optics (BX51, Olympus, Tokyo, Japan) and an infrared-sensitive video camera (Hamamatsu Photonics, Hamamatsu, Japan). The distance between Venus-positive and pyramidal cells was <100 μm. The electrical signals were recorded using amplifiers (Multiclamp 700B, Molecular Devices, Sunnyvale, CA, United States), digitized (Digidata 1440A, Molecular Devices) and observed on-line; the information was stored on a computer hard disk using Clampex software (pClamp 10, Molecular Devices).

The pipette solution used for recordings from interneurons and pyramidal cells contained the following (in mM): 70 potassium gluconate, 70 KCl, 10 HEPES, 15 biocytin, 0.5 EGTA, 2 MgCl_2_, 2 magnesium ATP, and 0.3 sodium GTP. The pipette solution had a pH of 7.3 and an osmolarity of 300 mOsm. The liquid junction potential for the current- and voltage-clamp recordings was -9 mV, and the voltage was corrected accordingly. Thin-wall borosilicate patch electrodes (2–5 MΩ) were pulled on a Flaming-Brown micropipette puller (P-97, Sutter Instruments, Novato, CA, United States).

The recordings were obtained at 30–31°C. The seal resistance was >5 GΩ, and only data obtained from electrodes with access resistance of 6–20 MΩ and <20% change during recordings were included in this study. The series resistance was 50% compensated. The membrane currents and potentials were low-pass filtered at 2–5 kHz and digitized at 20 kHz.

Before unitary excitatory/inhibitory postsynaptic current (uEPSC/uIPSC) or miniature EPSC (mEPSCs)/miniature IPSCs (mIPSCs) recordings, the voltage responses of pre- and postsynaptic cells were recorded after applying long hyperpolarizing and depolarizing current pulse (300 ms) injections to examine the basic electrophysiological properties, including input resistance, single spike kinetics, voltage-current relationships, repetitive firing patterns, and firing frequency.

### Multiple Whole-Cell Patch-Clamp Recording

Because some cell pairs had mutual or ≥2 connections, most cells were recorded under voltage-clamp conditions (holding potential = -70 mV) during uEPSC/uIPSC recordings. Short depolarizing voltage step pulses (2 ms, 80 mV) were applied to presynaptic cells to induce action currents. Where indicated, RAMH (Sigma–Aldrich, St. Louis, MO, United States) was added directly to the perfusate.

Before starting the successive recording of uEPSCs/uIPSCs, we checked the stability of their amplitude for 5–15 min, and excluded the connections that exhibited a gradual decrease of uEPSC/uIPSC amplitude.

Unitary events were recorded in normal ACSF for 5–10 min; we applied RAMH for 7.5–10 min, followed by washing for 10 min. To examine the blocking effect of H_3_ receptors, uEPSCs/uIPSCs were recorded under normal ACSF for at least 10 min, and JNJ 5207852 dihydrochloride (JNJ5207852; Tocris, Bristol, United Kingdom) or thioperamide maleate salt (Sigma–Aldrich), H_3_ receptor antagonists, was applied for 7.5 min. Subsequently, RAMH was applied in combination with JNJ5207852 or thioperamide for 7.5 min.

### Miniature EPSC and IPSC Recording

We recorded mEPSCs from layer V GABAergic interneurons under application of 1 μM tetrodotoxin (TTX) and 100 μM picrotoxin using the same internal pipette solution based on potassium gluconate and KCl as described above. The holding potential was -70 mV (Kobayashi et al., 2012).

Miniature IPSCs were recorded from layer V pyramidal cells under application of 1 μM TTX, 50 μM D-(-)-2-amino-5-phosphonopentanoic acid (D-APV), and 20 μM 6,7-dinitroquinoxaline-2,3-dione (DNQX). The internal pipette solution contained the following (in mM): 120 cesium gluconate, 20 biocytin, 10 HEPES, 8 NaCl, 5 *N*-ethyllidocaine chloride (QX-314), 2 magnesium ATP, 0.3 sodium GTP, and 0.1 BAPTA. The pipette solutions had a pH of 7.3 and an osmolarity of 300 mOsm. The holding potential was corrected for a calculated liquid junction potential of -12 mV and set at 0 mV.

The RAMH application protocol during mEPSC/mIPSC recording was same as that used for unitary event recording.

### Electrophysiological Data Analysis

Clampfit software (pClamp 10, Molecular Devices) was used to analyze the electrophysiological data. Input resistance was measured from slopes of least-squares regression lines fitted to *V-I* curves measured at the peak voltage deflection (current pulse amplitude up to -100 pA). By application of depolarizing step current pulses (300 ms), the action potential threshold was identified as the minimal potential from which the first action potential was elicited. The amplitude of action potential was measured from the resting membrane potential to the peak. The amplitude of AHP was measured from the negative peak to the action potential threshold. Repetitive firing in response to long (300 ms) depolarizing current pulses was evaluated by measuring the slope of least-squares regression lines in a plot of the number of spikes versus the amplitude of injected current (*F-I* curve; up to 250–300 pA). The amplitude of uIPSCs/uEPSCs was measured as the difference between the peak postsynaptic and baseline currents obtained during a 2–3 ms time window close to the onset. To measure the 20–80% rise time and decay time constants of uEPSCs/uIPSCs, single action currents were induced at 0.07–0.1 Hz, and 10–30 postsynaptic events were aligned to the peak of the presynaptic action currents and averaged.

To quantify the effect of RAMH on the amplitude, paired-pulse ratio (PPR) of the 2nd to 1st uEPSC/uIPSC amplitude, coefficient of variation (CV) and failure rate, we analyzed 15 events just before RAMH application and 225 s after drug application for control and RAMH data, respectively. The uEPSC/uIPSC amplitudes in the range of synaptic noise were considered as failures.

Miniature EPSCs/mIPSCs were detected at a threshold of three times the standard deviation of the baseline noise amplitude using event detection software (kindly provided by Dr. John Huguenard, Stanford University). To measure the amplitude, interevent interval, 20–80% rise time, half duration, and 80–20% decay time, mEPSCs/mIPSCs were analyzed from continuous 5 min recordings before and after RAMH application. To obtain cumulative plots of the interevent interval and the amplitude of mEPSCs/mIPSCs, >1000 events were sampled per each neuron (*n* = 11).

### Electron Microscopic Immunohistochemistry

Four male Sprague–Dawley rats (350–400 g) were used for immunohistochemistry. The rats were deeply anesthetized using sodium pentobarbital (80 mg/kg, i.p.) and transcardially perfused with 100 ml of heparinized normal saline, followed by 400 ml of a freshly prepared mixture of 4% paraformaldehyde and 0.001% glutaraldehyde in 0.1 M phosphate buffer (PB; pH 7.4). The IC was removed and postfixed in the same fixative for 2 h at 4°C. Transverse sections were cut on a microslicer (Vibratome, St. Louis, MO, United States) at 60 μm and cryoprotected in 30% sucrose in PB overnight at 4°C. The sections were frozen on dry ice for 20 mins and thawed in phosphate buffer saline (PBS; 0.01 M, pH 7.4) to enhance penetration. The sections were pretreated with 1% sodium borohydride for 30 min to quench the glutaraldehyde and blocked with 3% H_2_O_2_ for 10 mins to suppress endogenous peroxidases and 10% normal donkey serum (Jackson ImmunoResearch, West Grove, PA, United States) for 30 min to mask secondary antibody binding sites.

For double immunostaining for the H_3_ receptor and vesicular glutamate transporter 1 (VGLUT1) or glutamic acid decarboxylase (GAD) 65/67, the sections were incubated overnight in a mixture of rabbit anti-H_3_ receptor (1:50; AB5660P, Millipore, Billerica, MA, United States) and guinea pig anti-VGLUT1 (1:2,000; #135 304, Synaptic Systems, Göttingen, Germany) or mouse anti-GAD65/67 (1:1,000; MSA-225, Assay designs Stressgen, Ann Arbor, MI, United States) antibodies. After rinsing in PBS, the sections were incubated with a mixture of 1 nm gold-conjugated donkey anti-rabbit (1:50, EMS, Hatfield, PA, United States) and biotinylated donkey anti-guinea pig or biotinylated donkey anti-mouse (1:200, Jackson ImmunoResearch) antibodies for 2–3 h. The sections were postfixed with 1% glutaraldehyde in PB for 10 min, rinsed several times in PBS, incubated for 5 min in silver intensification solution (IntenSE^TM^ M, Amersham, Arlington Heights, IL, United States), and rinsed in 0.1 M sodium acetate and PB. Subsequently, the sections were incubated with ExtrAvidin peroxidase (1:5,000, Sigma, St. Louis, MO, United States) for 1 h, and the immunoperoxidase was visualized using nickel-intensified 3,3′-diaminobenzidine tetrahydrochloride (DAB). The sections were further rinsed in PB, osmicated (0.5% osmium tetroxide in PB) for 30 min, dehydrated in graded alcohols, flat-embedded in Durcupan ACM (Fluka, Buchs, Switzerland) between strips of Aclar plastic film (EMS), and cured for 48 h at 60°C. Chips containing immunoreactivity for the H_3_ receptor and VGLUT1 or GAD65/67 in the dysgranular IC region of the IC were cut from the wafers and glued onto blank resin blocks using cyanoacrylate. Thin sections were collected on Formvar-coated single-slot nickel grids and stained with uranyl acetate and lead citrate.

The grids were examined on a Hitachi H 7500 electron microscope (Hitachi, Tokyo, Japan) at 80 kV accelerating voltage. The images were captured using Digital Montage software driving a MultiScan cooled CCD camera (ES1000W, Gatan, Pleasanton, CA, United States) attached to the microscope and saved as TIFF files. To control for the specificity of primary antibodies, the sections were processed according to the above protocol, except that the primary or secondary antibodies were omitted to completely abolish specific staining.

### *In Vivo* Optical Imaging

The method of optical imaging with a voltage-sensitive dye has been described previously (Fujita et al., 2010, 2011, 2012, 2017; Mizoguchi et al., 2011), and only a brief account of the methods employed will be given here. Five-week-old male Sprague–Dawley rats (Sankyo Labo, Tokyo, Japan) weighing 152.9 ± 4.5 g (*n* = 12) received atropine methyl bromide (5 mg/kg, i.p.) and were anesthetized with urethane (1.5 g/kg, i.p.). Anesthesia was maintained throughout the experiments by injecting additional urethane. The adequacy of anesthesia was gauged by the absence of the toe pinch reflex. Animals received a tracheotomy and intubation, and lidocaine (2% gel) was applied to the incisions to ensure complete analgesia. The anesthetized animal was mounted on a custom-made stereotaxic snout frame (Narishige, Tokyo, Japan), which was then tilted 60° laterally to make the left IC accessible to the CCD camera system and electrodes. The left temporal muscle and zygomatic arch were carefully removed, and a craniotomy was performed to expose the IC and the surrounding cortices.

The voltage-sensitive dye, RH1691 (1 mg/ml, Optical Imaging, New York, NY, United States) in 0.9% saline, was applied to the cortical surface for approximately 1 h, residual dye was rinsed with saline for approximately 30 min, and fluorescent changes of RH1691 were measured by a CCD camera system (MiCAM02, Brainvision, Tokyo, Japan) mounted on a stereomicroscope (Leica Microsystems, Wetzlar, Germany). The cortical surface was illuminated through a 632 nm excitation filter and a dichroic mirror by a tungsten-halogen lamp (CLS150XD, Leica Microsystems), and the fluorescent emission was captured through an absorption filter (λ > 650 nm long-pass, Andover, Salem, NH, United States). The CCD-based camera had a 6.4 mm^2^ × 4.8 mm^2^ imaging area consisting of 184 pixels × 124 pixels. To eliminate signal due to acute bleaching of the dye, the final image was obtained by subtracting an image without stimulation from an image with stimulation. The sampling interval was 4 ms. The acquisition time was set at 500 ms. Sixteen consecutive images in response to stimuli were averaged to reduce noise, including interference from the heartbeat, breathing, and spontaneous neural activity.

A tungsten concentric bipolar electrode (impedance = 500 KΩ; Unique Medical, Tokyo, Japan) was inserted 0.3 mm from the surface of the IC. The duration and intensity of stimulation pulse was set at 100 μs and 7 V. Five train pulses with a 50 ms interstimulus interval were applied. Each train pulse was delivered at 30 s intervals. After optical recording in control, RAMH (10 μM) was applied to the cortical surface for 60 min. Another optical recording was performed during the last 30 min of RAMH application.

The change in the intensity of fluorescence (Δ*F*) in each pixel, relative to the initial fluorescence intensity (*F*), was calculated (Δ*F*/*F*). For the measurement of amplitude and excitatory area, images of responses were processed with a spatial filter (9 pixels × 9 pixels) to avoid the influence of noise and brain movements on each pixel. A significant response was defined as a signal exceeding seven times the SD of the baseline noise. Optical imaging data were processed and represented using software (Brain Vision Analyzer, Brainvision, Tokyo, Japan). To quantify the spatiotemporal profiles of excitation, we set a region of interest (ROI) in the adjacent region of the stimulation site.

All chemicals, unless otherwise specified, were purchased from Sigma–Aldrich.

### Statistics

The data are presented as the means ± SEM. Comparisons of the uEPSC/uIPSC amplitude and PPR between control and drug application were conducted using the paired *t*-test. The suppression rates of uEPSCs in pyramidal cell to pyramidal cell (Pyr→Pyr) or Pyr→GABAergic interneuron (Interneuron) connections, and uIPSCs in Interneuron→Pyr connections were analyzed using one-way ANOVA followed by *post hoc* Bonferroni test. Reductions in the amplitude of uEPSC/uIPSCs induced through five trains or paired-pulses were compared using Student’s *t*-test. Failure rate was compared between control and RAMH using Wilcoxon signed-rank test. Box-and-whisker plots were made as follows: upper and lower limit of box indicates 75th and 25th percentile, respectively; line within box denotes the median; upper and lower limits of the capped lines indicate 90th and 10th percentile, respectively. The RAMH-induced suppression rates of uIPSC amplitude were compared between the 1st and the 2nd–5th uIPSCs using two-tailed multiple *t*-tests with Bonferroni correction. The amplitude and interevent interval of mEPSCs/mIPSCs were analyzed through non-parametric statistical analysis (Kolmogorov–Smirnoff test; K-S test) to assess the significance of the shifts in the cumulative probability distributions in control and RAMH application. Paired *t*-tests were used to compare the mean amplitude and interevent interval of mEPSCs/mIPSCs and the mean amplitude of excitation (Δ*F/F*) and excitation area in the optical imaging study. A level of *P* < 0.05 indicated statistical significance.

## Results

The IC is a rostrocaudally extended region that lies on the dorsal surface of the rhinal fissure. The results obtained in the present study comprised recordings obtained from granular and dysgranular IC neurons around the middle cerebral artery, which are part of the gustatory cortex (Yamamoto et al., 1980; Accolla et al., 2007; Kobayashi et al., 2010).

To explore the roles of H_3_ receptors in the regulation of local circuits in the IC, we performed paired whole-cell patch-clamp recording from pyramidal cells and GABAergic neurons. VGAT-Venus line A transgenic rats were used to identify GABAergic neurons. In this study, GABAergic neurons in the IC were divided into fast spiking (FS) and non-FS neurons, which included late spiking and regular spiking neurons (Kawaguchi and Kubota, 1997; Koyanagi et al., 2010, 2014; Yamamoto et al., 2010, 2015; Kobayashi, 2011; Kobayashi et al., 2012). **Table 1** summarizes the basic membrane properties of the recorded neurons. Before examining the synaptic connections between recording cells, we classified the recording GABAergic neurons into FS and non-FS neurons after the application of long depolarizing current pulses under current clamp conditions. Based on the results, the cell connection subtypes were classified as (1) pyramidal cell to pyramidal cell (Pyr→Pyr), (2) Pyr→FS, (3) Pyr→non-FS, (4) FS→Pyr, and (5) non-FS→Pyr. The present study did not examine connections between GABAergic neurons.

**Table 1:** Intrinsic electrophysiological properties of insular cortical neurons.

Neuron subtype	Pyramidal Mean ± SEM	Fast spiking Mean ± SEM	Regular spiking Mean ± SEM	Late spiking Mean ± SEM
The number of neurons	66	35	10	11
Vm^a^ (mV)	-72.8 ± 1.0	-73.6 ± 1.2	-67.6 ± 2.7	-74.0 ± 1.8
Input resistance (MΩ)	167.8 ± 8.7	176.9 ± 19.2	232.5 ± 33.7	232.5 ± 33.7
**Action potential**				
Threshold (mV)	50.5 ± 0.5	-50.9 ± 0.5	50.7 ± 0.9	48.5 ± 0.9
Amplitude (mV)	94.2 ± 1.8	76.2 ± 2.2***	82.4 ± 5.3	82.4 ± 2.8*
Half duration (ms)	1.63 ± 0.06	0.76 ± 0.04***	1.3 ± 0.1###	0.96 ± 0.10***
AHP^b^ amplitude (mV)	-11.0 ± 0.5	-18.8 ± 0.71***	-11.4 ± 1.9###	-18.2 ± 1.4***,^††^
Rheobase (pA)	82.0 ± 10.7	156.0 ± 11.7***	75.0 ± 28.4#	131.8 ± 24.0
**Repetitive firing**				
*F-I* slope^c^ (spikes/pA)	0.05 ± 0.01	0.19 ± 0.02***	0.07 ± 0.01##	0.14 ± 0.01


*^a^Resting membrane potential; ^b^Afterhyperpolarization. One-way ANOVA followed by *post hoc* Bonferroni’s test. ^c^The number of spikes for 300 ms is divided by the intensity of the injected current (pA). ^∗^, ^∗∗∗^*P* < 0.05, 0.001 in comparison with pyramidal cell. ^#^, ^##^, ^###^*P* < 0.05, 0.01, 0.001 in comparison with fast spiking cell. ^††^*P* < 0.01 in comparison with regular spiking cell.*

### H_3_ Receptor Activation Suppresses uEPSCs Obtained from Pyr→Pyr and Pyr→FS/Non-FS Connections

**FIGURE 1:**
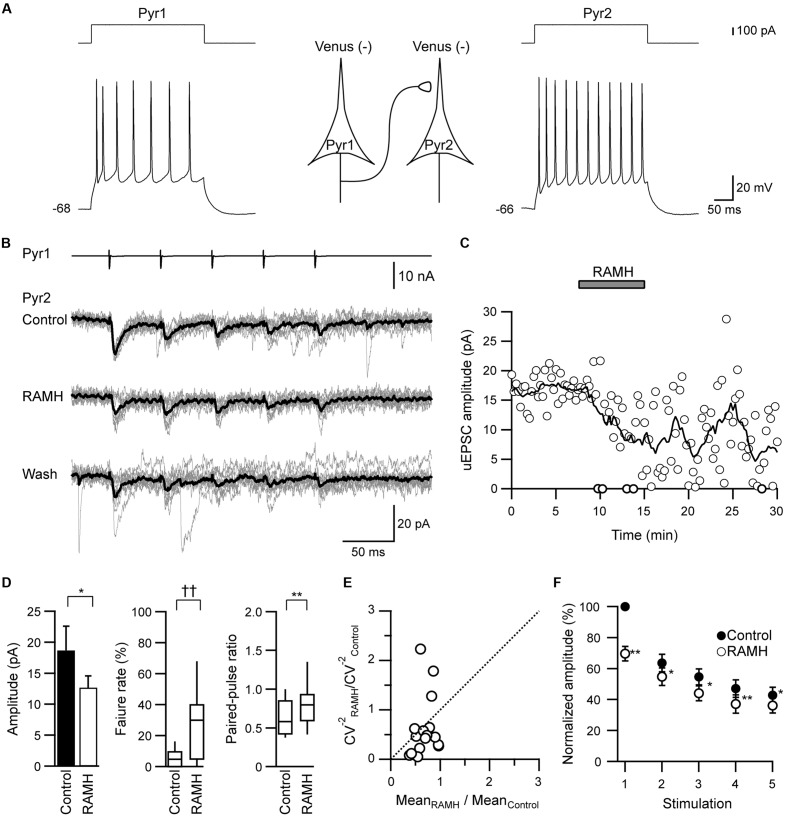
The effects of R-α-methylhistamine (RAMH) on unitary excitatory postsynaptic currents (uEPSCs) in pyramidal cell to pyramidal cell (Pyr→Pyr) connections. **(A)** A scheme of a Pyr→Pyr connection (Pyr1→Pyr2) with suprathreshold voltage responses of each Pyr (bottom traces) in response to depolarizing current pulse injection (upper traces). The resting membrane potentials are shown to the left of the traces. **(B)** uEPSC recordings from the pyramidal cell (Pyr2) responding to an injection of five short train pulses to the pyramidal cell (Pyr1; 2 ms, +80 mV, 20 Hz; upper traces) shown in **(A)** (Vh = –70 mV). Eleven consecutive traces (gray) and their average traces (black) are shown. Note that the uEPSC amplitude was reduced through the application of 10 μM RAMH. **(C)** Time course of the uEPSC amplitude before, during, and after 10 μM RAMH application in Pyr→Pyr connections shown in **(A,B)**. **(D)** RAMH-induced effects on the uEPSC amplitude, failure rate, and paired-pulse ratio in Pyr→Pyr connections (*n* = 17). **(E)** Coefficient of variation (CV) analysis in Pyr→Pyr connections. The inverse of the square of CV for the 1st uEPSC amplitude under the application of RAMH is plotted against the mean amplitude; both CV and mean are normalized to the respective values of the 1st uEPSCs in controls. **(F)** The normalized amplitude of the 1st to 5th uEPSCs (*n* = 17). Filled and open circles indicate the normalized amplitude of uEPSCs in control and during RAMH application, respectively. ^∗^*P* < 0.05, ^∗∗^*P* < 0.01, paired *t*-test. ^††^*P* < 0.01, Wilcoxon test.

To examine the H_3_ receptor-mediated effects on the excitatory synaptic connections (Pyr→Pyr, Pyr→FS, and Pyr→non-FS), 10 μM RAMH, an H_3_ receptor agonist, was applied to the perfusate. The pyramidal cells were identified among Venus-negative pyramidal somata after repetitive firing with spike adaptations (**Figure 1A**).

In Pyr→Pyr connections, the bath application of RAMH (10 μM) invariably suppressed the 1st uEPSC amplitude by 30.4 ± 4.6% (*n* = 17; *P* < 0.05, paired *t*-test; **Figures 1B–D**), which was not recovered after a 10 min washout (**Figure 1C**). The RAMH-induced uEPSC suppression was accompanied by increases in the failure rate of the 1st uEPSCs (*n* = 17; *P* < 0.01, Wilcoxon test) and PPR (*n* = 17; *P* < 0.01, paired *t*-test), as shown in **Figure 1D**. In these Pyr→Pyr connections, the 2nd to 5th uEPSCs were less affected after the application of RAMH (**Figure 1E**), suggesting that the effect of RAMH is likely to be mediated by a presynaptic mechanism.

**FIGURE 2:**
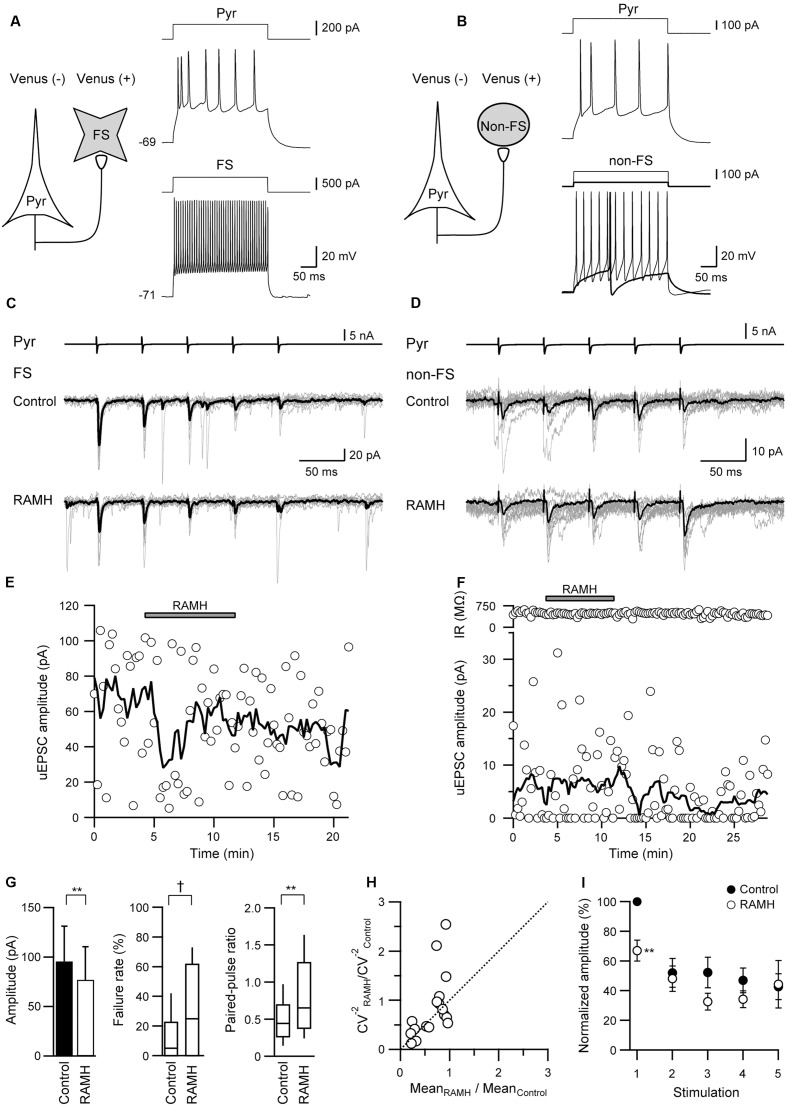
The effects of 10 μM RAMH on uEPSCs in pyramidal cell to fast-spiking (FS) or non-fast-spiking neuron (non-FS; Pyr→FS/non-FS) connections. **(A,B)** Schemes of a Pyr→FS **(A)** and a Pyr→non-FS connection **(B)** with suprathreshold voltage responses of each neuron. **(C,D)** uEPSC recordings from the Pyr→FS **(C)** and the Pyr→non-FS **(D)** connections responding to an injection of five short train pulses to the pyramidal cells (2 ms, +80 mV, 20 Hz; upper traces) shown in **(A,B)**, respectively (Vh = –70 mV). Eleven consecutive traces (gray) and their average traces (black) are shown. Note that the uEPSC amplitude was reduced after RAMH application in both connections. **(E**,**F)** Time courses of the uEPSC amplitude before, during, and after RAMH application in Pyr→FS **(E)** and Pyr→non-FS connections **(F)**. Input resistance (IR) was shown in **(F)**. **(G)** RAMH-induced effects on the uEPSC amplitude, failure rate, and paired-pulse ratio in Pyr→FS/non-FS connections (*n* = 18). **(H)** CV analysis in Pyr→FS/non-FS connections. **(I)** The normalized amplitude of the 1st to 5th uEPSCs (*n* = 18). Filled and open circles indicate the normalized amplitude of uEPSCs in control and during RAMH application, respectively. ^∗^*P* < 0.05, ^∗∗^*P* < 0.01, paired *t*-test. ^†^*P* < 0.05, Wilcoxon test.

In the Pyr→FS connections, 10 μM RAMH invariably suppressed the 1st uEPSC amplitude by 30.5 ± 6.7% (*n* = 12; *P* < 0.01, paired *t*-test; **Figures 2A,C**). Similarly, Pyr→non-FS connections showed consistent suppression of the 1st uEPSC amplitude by 52.9 ± 12.8% (*n* = 6; *P* < 0.05, paired *t*-test; **Figures 2B,D**). The RAMH-induced suppression of uEPSCs did not recover after the 10 min washout (**Figures 2E,F**). As there was no significant difference between the reduction rates in the 1st uEPSC amplitude (*P* = 0.19, Student’s *t*-test), we combined the results obtained from Pyr→FS and Pyr→non-FS connections into one category, Pyr→Interneuron. Generally, RAMH suppressed the 1st uEPSC amplitude by 37.9 ± 6.6% (*n* = 18; *P* < 0.01, paired *t*-test), which was comparable to that observed in Pyr→Pyr connections (*P* = 0.37, Student’s test). The RAMH-induced uEPSC suppression was accompanied by increases in the failure rate of the 1st uEPSCs (*n* = 18; *P* < 0.05, Wilcoxon test) and PPR (*n* = 18; *P* < 0.01, paired *t*-test), as shown in **Figure 2G**.

In contrast to the smaller uEPSC amplitude in response to the 2nd to 5th presynaptic action currents in Pyr→Pyr connections (**Figure 1F**), Pyr→Interneuron connections frequently showed paired-pulse facilitation. RAMH had little effect on the 2nd to 5th uEPSC amplitude in Pyr→Interneuron connections (*n* = 18; *P* > 0.1, paired *t*-test; **Figure 2I**).

RAMH did not induce a significant change in input resistance in Pyr (*n* = 90, *P* = 0.79, paired *t*-test), FS (*n* = 30, *P* = 0.85, paired *t*-test), and non-FS neurons (*n* = 8, *P* = 0.91, paired *t*-test).

These results suggest that a decrease in uEPSCs through RAMH might reflect presynaptic modulation via H_3_ receptors.

Coefficient of variation analysis is another method to evaluate the synaptic efficacy changes occur at pre- or postsynaptic sites (Faber and Korn, 1991; Arami et al., 2013). According to a binomial model of synaptic transmission, functional changes in presynaptic sites are expected to be accompanied by a change in the CV of synaptic responses: Presynaptic changes result in the plot that CV^-2^ values against the efficacy changes are located on or below the diagonal line in the case of low release probability. Our CV analysis show in **Figures 1E**, **2H** supports the hypothesis that the suppressive effect of RAMH on uEPSCS is likely to be mediated via a presynaptic mechanism.

### H_3_ Receptor Activation Suppresses uIPSCs Obtained from FS/Non-FS→Pyr Connections

**FIGURE 3:**
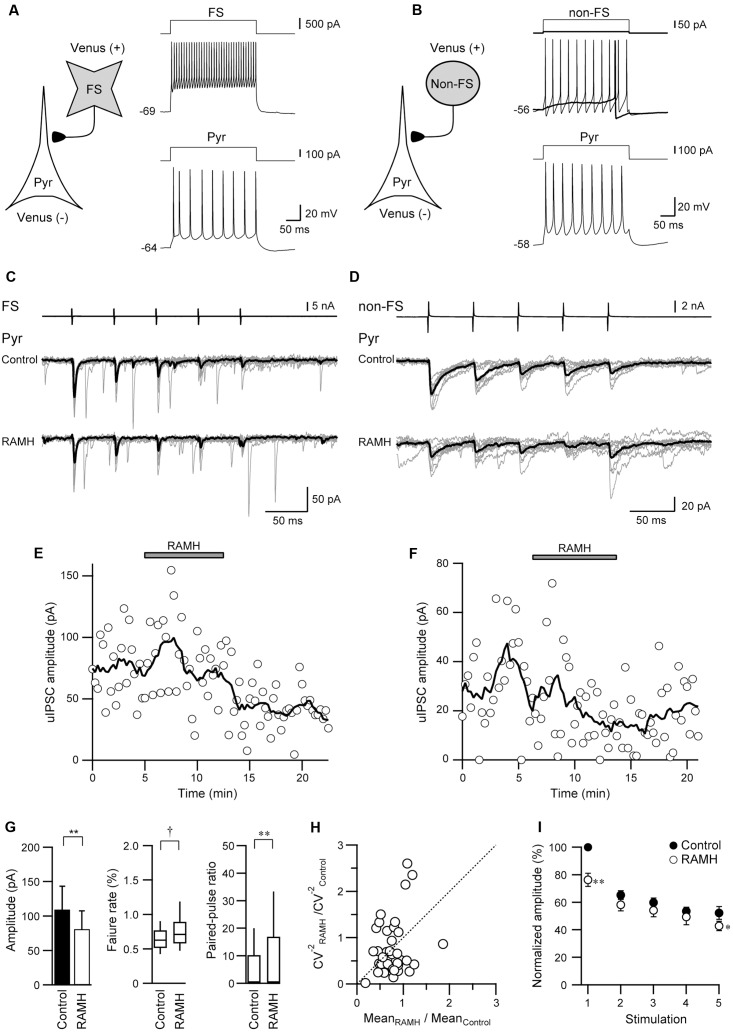
The effects of 10 μM RAMH on uIPSCs in FS or non-FS neuron to pyramidal cell (FS→Pyr and non-FS→Pyr) connections. **(A,B)** Schemes of a FS→Pyr **(A)** and a non-FS→Pyr connection **(B)** with suprathreshold voltage responses of each neuron. **(C,D)** uIPSC recordings from the FS→Pyr **(C)** and the non-FS→Pyr **(D)** connections responding to an injection of five short train pulses to the FS/non-FS (2 ms, +80 mV, 20 Hz; upper traces) shown in **(A,B)**, respectively. Eleven consecutive traces (gray) and their average traces (black) are shown. Note that the uIPSC amplitude was reduced after RAMH application in both connections. **(E,F)** Time courses of the uIPSC amplitude before, during, and after RAMH application in Pyr→FS **(E)** Pyr→non-FS **(F)** connections. **(G)** RAMH-induced effects on the uEPSC amplitude, failure rate, and paired-pulse ratio in FS/non-FS→Pyr connections (*n* = 39). **(H)** CV analysis in FS/non-FS→Pyr connections. **(I)** The normalized amplitude of the 1st to 5th uIPSCs (*n* = 33). Filled and open circles indicate the normalized amplitude of uIPSCs in control and during RAMH application, respectively. ^∗^*P* < 0.05, ^∗∗^*P* < 0.01, paired *t*-test. ^†^*P* < 0.05, Wilcoxon test.

**Figure 3** shows a typical example of the effects of RAMH on uIPSCs obtained from FS→Pyr (**Figures 3A,C**) and non-FS→Pyr (**Figures 3B,D**) connections. In both pairs, 10 μM RAMH almost consistently suppressed the amplitude of uIPSCs. RAMH reduced the 1st uIPSC amplitude by 20.0 ± 5.3% in FS→Pyr connections (*n* = 31; *P* < 0.01, paired *t*-test) and 35.7 ± 9.6% in non-FS→Pyr connections (*n* = 8; *P* < 0.05, paired *t*-test). **Figures 3E,F** are the time courses of uIPSC amplitude before, during and after the application of RAMH in FS→Pyr and non-FS→Pyr shown in **Figures 3A–D**, respectively. The recovery of uIPSC amplitude was not observed after a 10 min washout.

As there was no significant difference between these reduction rates (*P* = 0.11, Student’s *t*-test), we combined the results obtained from FS→ and non-FS→Pyr synapses into one category, Interneuron→Pyr. Generally, RAMH suppressed uIPSC amplitude by 23.0 ± 4.7% (*n* = 39; *P* < 0.01, paired *t*-test), which was almost comparable to that of the uEPSC suppression observed in Pyr→Pyr or Pyr→Interneuron connections (*P* = 0.19, one-way ANOVA).

Similar to Pyr→Interneuron connections, H_3_ receptors likely suppress uIPSC amplitudes via a presynaptic mechanism. The RAMH-induced uIPSC suppression was accompanied by increases in the failure rate of the 1st uIPSCs (*n* = 39; *P* < 0.05, Wilcoxon test) and PPR (*n* = 39; *P* < 0.05, paired *t*-test), as shown in **Figure 3G**. The result of CV analysis (**Figure 3H**) also suggested that the suppressive effect of RAMH on uIPSCS is induced by a presynaptic mechanism. Interneuron→Pyr connections exhibited paired-pulse depression in the control, and the analysis of the 2nd and 5th uEPSC amplitude showed a significant suppression after the application of RAMH (*n* = 33; *P* < 0.05, paired *t*-test; **Figure 3I**). The RAMH-induced suppression rate of the 1st uIPSC amplitude was significantly larger than those of the 2nd and 5th uIPSCs (*P* < 0.001; two-tailed multiple *t*-test with Bonferroni correction), suggesting that the effect of RAMH is likely to be mediated by a presynaptic mechanism.

These results suggest that H_3_ receptors suppress the uIPSC amplitude between GABAergic and pyramidal cells in the IC, which is likely mediated via a presynaptic mechanism, i.e., the suppression of GABA release from FS and non-FS cells to pyramidal cells.

### An H_3_ Receptor Antagonist Increases the uIPSC Amplitude

**FIGURE 4:**
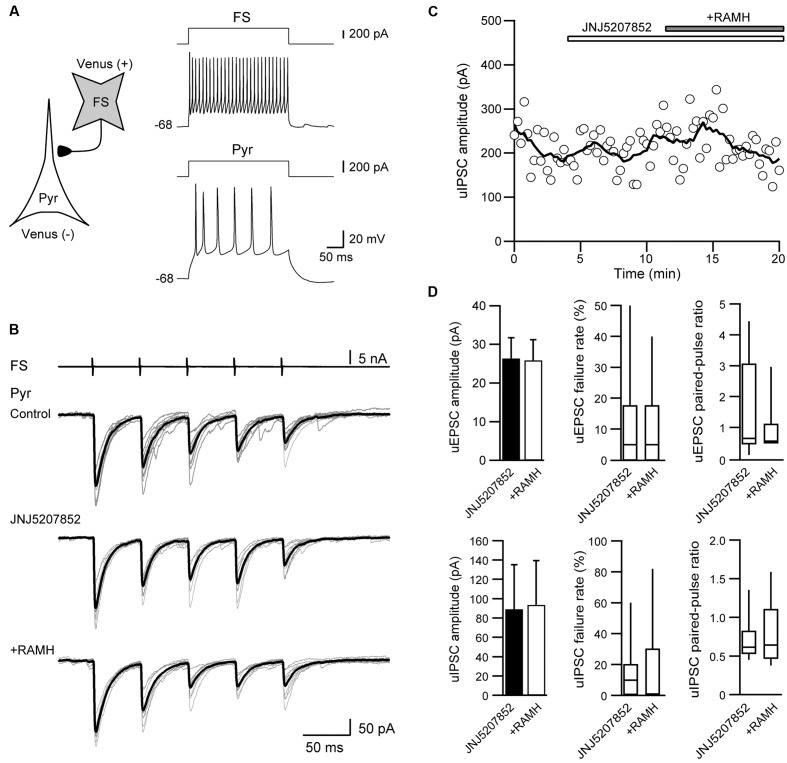
JNJ5207852 (10 μM), an H_3_ receptor antagonist, blocks RAMH-induced suppression of uIPSCs in FS/non-FS→Pyr connections. **(A)** A scheme of a FS→Pyr connection with suprathreshold voltage responses of FS and Pyr cells in response to depolarizing current pulse injection. **(B)** uIPSC recordings from the pyramidal cell in response to an injection of five short train pulses to the FS (2 ms, +80 mV, 20 Hz; upper traces) shown in **(A)** (Vh = –70 mV). Eleven consecutive traces (gray) and their average traces (black) are shown. RAMH (10 μM) had little effect on the uIPSC amplitude under application of JNJ5207852. **(C)** Time course of the uIPSC amplitude. The periods of JNJ5207852 and RAMH application are shown on the top of the graph. **(D)** The slight effect of RAMH on the uEPSC (*n* = 8) and uIPSC amplitude under application of JNJ5207852 (*n* = 13).

To confirm that the suppression of uIPSCs through RAMH is mediated via H_3_ receptors, we examined the effects of the pre-application of JNJ5207852, an H_3_ receptor antagonist, on the RAMH-induced suppression of uIPSCs. **Figures 4A–C** showed a typical example of the effects of the pre-application of 10 μM JNJ5207852 followed by 10 μM RAMH on uIPSCs obtained from FS→Pyr connections. Application of JNJ5207852 itself had little effect both on the amplitude of uEPSCs obtained from Pyr→Pyr/Interneuron connections (38.6 ± 8.8 pA to 26.4 ± 5.3 pA, *n* = 8, *P* = 0.14, paired *t*-test) and uIPSCs in Interneuron→Pyr connections (88.8 ± 46.5 pA to 89.3 ± 45.9 pA, *n* = 13, *P* = 0.15, paired *t*-test). RAMH had little effect of the amplitude of Pyr→Pyr/Interneuron connections under application of JNJ5207852 (26.4 ± 5.3 pA to 25.9 ± 5.3 pA, *n* = 8; **Figure 4D**). Similarly, in 13 Interneuron→Pyr connections, RAMH (10 μM), in combination with JNJ5207852 (10 μM), had little effect on the amplitude of uIPSCs (89.3 ± 45.9 pA to 93.8 ± 45.7 pA; *n* = 13, *P* = 0.66, paired *t*-test; **Figure 4D**). These results suggest that the RAMH-induced suppression of uEPSCs and uIPSCs is induced through H_3_ receptors.

### Thioperamide Increases the uIPSC Amplitude

**FIGURE 5:**
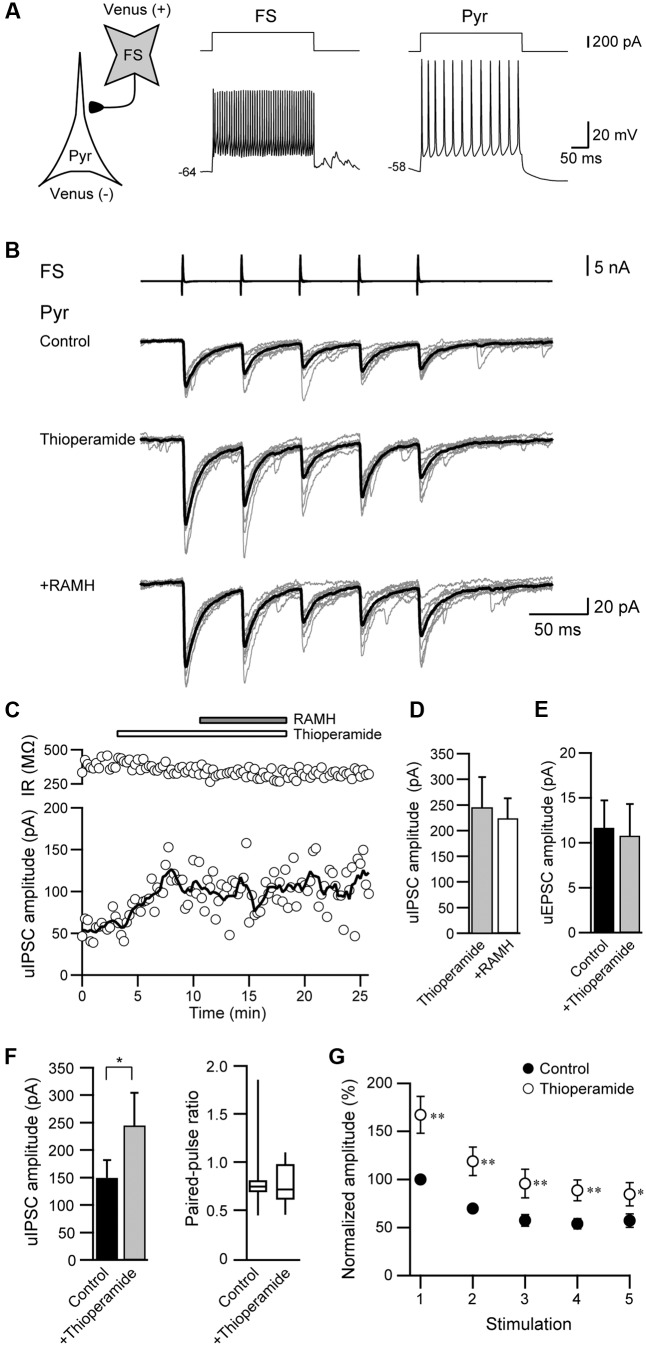
Thioperamide (10 μM), an H_3_ receptor antagonist, blocks RAMH-induced suppression of uIPSCs in FS/non-FS→Pyr connections. **(A)** A scheme of a FS→Pyr connection with suprathreshold voltage responses of FS and Pyr cells in response to depolarizing current pulse injection. **(B)** uIPSC recordings from the pyramidal cell in response to an injection of five short train pulses to the FS (2 ms, +80 mV, 20 Hz; upper traces) shown in **(A)** (Vh = –70 mV). Eleven consecutive traces (gray) and their average traces (black) are shown. RAMH (10 μM) had little effect on the uIPSC amplitude under application of thioperamide. Note that a slight increase of the uIPSC amplitude was induced through the application of thioperamide alone. **(C)** Time course of the uIPSC amplitude and IR. The periods of thioperamide and RAMH application are shown on the top of the graph. **(D)** The slight effect of RAMH on the uIPSC amplitude under application of thioperamide (*n* = 14). **(E)** The effects of thioperamide on the uEPSC (*n* = 5). **(F)** The effects of thioperamide on the uIPSC amplitude (*n* = 10). Note the significant increase in uIPSC amplitude after thioperamide application without changes in the failure rate and paired-pulse ratio of uIPSCs. **(G)** The normalized amplitude of the 1st to 5th uIPSCs (*n* = 10). Filled and open circles indicate the normalized amplitude of uIPSCs in control and during thioperamide application, respectively. ^∗^*P* < 0.05, ^∗∗^*P* < 0.01, paired *t*-test.

We also examined the effects of thioperamide, an H_3_ receptor antagonist, which is known as an inverse agonist (Morisset et al., 2000). **Figures 5A–C** showed a typical example of the effects of the pre-application of 10 μM thioperamide followed by 10 μM RAMH on uIPSCs obtained from FS→Pyr connections. Under thioperamide application, RAMH had little effect on the uIPSC amplitude. In 14 Interneuron→Pyr connections, RAMH (10 μM), in combination with thioperamide (10 μM), had little effect on the amplitude of uIPSCs (244.9 ± 59.7 pA to 224.4 ± 38.7 pA; *n* = 8, *P* = 0.48, paired *t*-test; **Figure 5D**). Similarly, RAMH also showed little effect of the amplitude of Pyr→Pyr connections under application of thioperamide (14.7 ± 6.8 pA to 15.0 ± 7.3 pA, *n* = 2). These results indicated that the RAMH-induced suppression of uIPSC amplitudes was mediated through H_3_ receptors.

In addition to the blockade of RAMH-induced suppression of uIPSCs by thioperamide, we observed that the application of thioperamide alone increased the uIPSC amplitude compared with the control (**Figures 5B,C**), though thioperamide had no significant effect on uEPSCs in Pyr→Pyr connections (*n* = 5; *P* = 0.58, paired *t*-test; **Figure 5E**). In 10 Interneuron→Pyr connections, thioperamide (10 μM) significantly increased the amplitude of uIPSCs by 102.3 ± 37.1% (*P* < 0.05, paired *t*-test; **Figure 5F**). The enhancement of uIPSCs through thioperamide application was not accompanied by significant changes in the failure rate of the 1st uIPSCs (*n* = 10, *P* = 0.18, Wilcoxon test) and the PPR (*n* = 10; *P* = 0.57, paired *t*-test; **Figure 5F**), and the facilitative effect of thioperamide was larger in the 1st uIPSCs compared with the 3rd uIPSCs (*P* < 0.05; two-tailed multiple *t*-test with Bonferroni correction; **Figure 5G**).

Thioperamide did not induce a significant change in input resistance in Pyr (*n* = 37, *P* = 0.95, paired *t*-test) and FS (*n* = 10, *P* = 0.96, paired *t*-test).

These results suggest that thioperamide facilitates GABAergic synaptic transmission via postsynaptic mechanisms in the connection of Interneuron→Pyr.

### mEPSC and mIPSC Frequencies Are Suppressed through an H_3_ Receptor Agonist

The frequency of synaptic events under the application of the Na^+^ channel blocker, TTX, reflects the release probability of neurotransmitters, including GABA and glutamate. To strengthen the hypothesis that H_3_ receptors suppress GABA/glutamate release via presynaptic H_3_ heteroreceptors, mEPSCs and mIPSCs were recorded from pyramidal cells and GABAergic interneurons, respectively, under the application of TTX and glutamatergic/GABAergic receptor antagonists.

**FIGURE 6:**
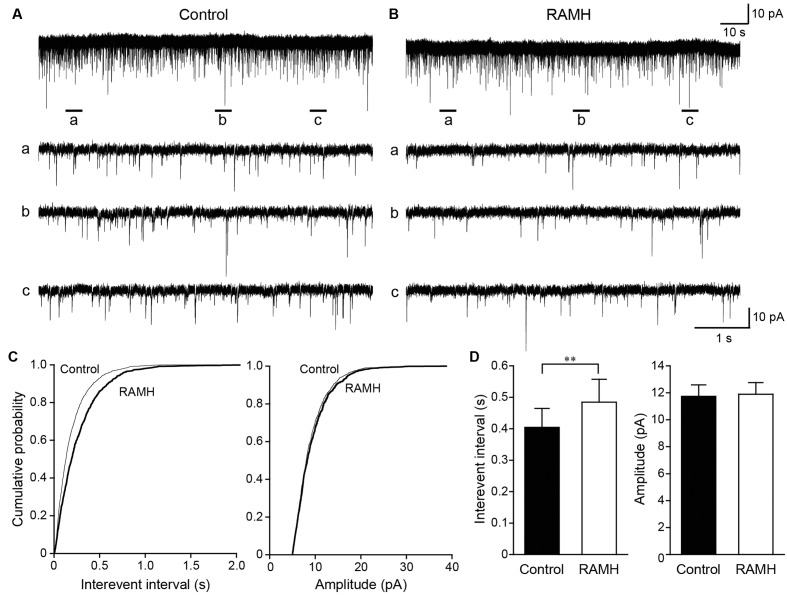
The effects of 10 μM RAMH on miniature EPSCs (mEPSCs) recorded from insular cortex (IC) layer V pyramidal cells under the application of 1 μM TTX and 10 μM bicuculline. **(A,B)** An example of mEPSCs recorded before **(A)** and during the application of RAMH **(B)**. The holding potential was set at –70 mV. Bottom panels **(a–c)** are time-expanded views of the regions indicated with bars under the top trace. **(C)** Cumulative probability plots of the interevent interval (left) and the amplitude of the mEPSCs (right) shown in **(A,B)**. Note that RAMH (thick black lines) increased the interevent interval of mEPSCs without changing the amplitude. **(D)** Mean interevent interval (left) and the amplitude of mEPSCs (right) in controls and during RAMH application. RAMH increased the mIPSC interevent interval (^∗∗^*P* < 0.01, paired *t*-test).

**Figure 6** shows the typical effect of RAMH on mEPSCs obtained from GABAergic interneurons in the layer V IC. In addition, 100 μM of picrotoxin was applied to block GABA_A_ receptor-dependent currents. The interevent interval of mEPSCs was reduced through RAMH (**Figures 6A–C**; *P* < 0.001; K-S test) without changing the amplitude (**Figures 6A–C**; *P* = 0.16; K-S test). A total of 11 neurons were analyzed to compare the mean interevent interval and amplitude of mEPSCs recorded under control and RAMH conditions. RAMH significantly increased the interevent interval of mIPSCs (0.40 ± 0.06 s vs. 0.48 ± 0.07 s, *n* = 11, *P* < 0.01, paired *t*-test) without affecting the mIPSC amplitude (11.7 ± 0.9 pA vs. 11.9 ± 0.9 pA, n = 11, *P* = 0.66, paired *t*-test; **Figure 6D**).

**FIGURE 7:**
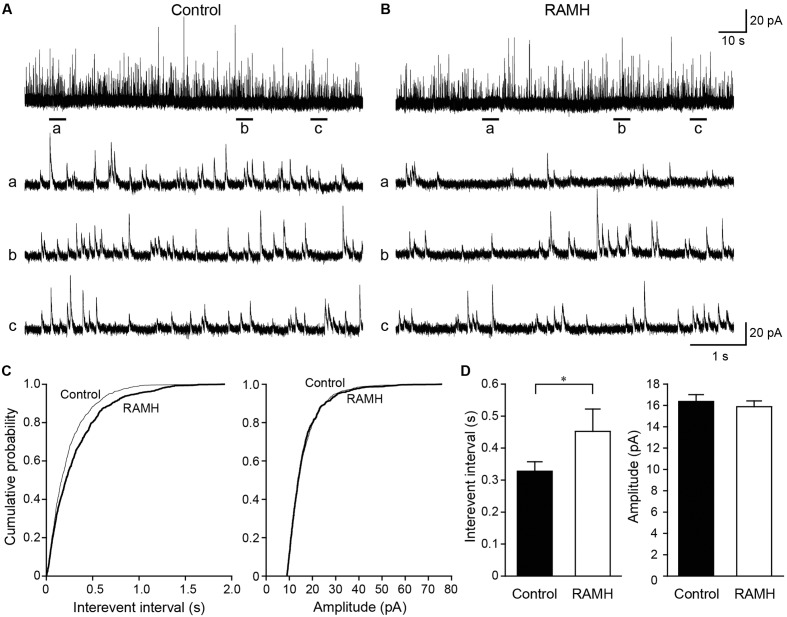
The effects of 10 μM RAMH on miniature IPSCs (mIPSCs) recorded from the IC layer V pyramidal cells under the application of 1 μM TTX, 50 μM D-(-)-2-amino-5-phosphonopentanoic acid (D-APV), and 20 μM 6,7-dinitroquinoxaline-2,3-dione (DNQX). **(A,B)** An example of mIPSCs recorded before **(A)** and during the application of RAMH **(B)**. The holding potential was set at 0 mV. Bottom panels **(a–c)** are time-expanded views of the regions indicated with bars under the top trace. **(C)** Cumulative probability plots of the interevent interval (left) and the amplitude of the mIPSCs (right) shown in **(A,B)**. Note that RAMH (thick black lines) increased the interevent interval of mIPSCs without changing their amplitude. **(D)** Mean interevent interval (left) and the amplitude of mIPSCs (right) in controls and during RAMH application. RAMH increased the mIPSC interevent interval (^∗^*P* < 0.05, paired *t*-test).

We recorded mIPSCs from layer V IC pyramidal cells under the application of TTX (1 μM), D-APV (50 μM), and DNQX (20 μM; **Figures 7A–C**). Similar to mEPSCs, RAMH (10 μM) reduced the frequency of mIPSCs (**Figures 7A,B**). A cumulative probability plot obtained from the same neuron indicates that RAMH significantly increased the interevent interval of mIPSCs (*P* < 0.001; K-S test; **Figure 7C**). However, RAMH did not affect the amplitude of mIPSCs (*P* = 0.51; K-S test; **Figure 7C**). A total of 11 neurons were analyzed to compare the mean interevent interval and amplitude of mIPSCs recorded under control and RAMH conditions. RAMH significantly increased the interevent interval of mIPSCs (0.33 ± 0.03 s vs. 0.45 ± 0.07 s, *n* = 11, *P* < 0.05, paired *t*-test) without affecting the mIPSC amplitude (16.4 ± 0.6 pA vs. 15.9 ± 0.6 pA, *n* = 11, *P* = 0.12, paired *t*-test; **Figure 7D**).

These miniature recordings support the hypothesis derived from the results of unitary synaptic event recordings, suggesting that H_3_ receptors in glutamatergic and GABAergic terminals regulate the release of glutamate and GABA, respectively.

### Electron Microscopy and Immunohistochemistry

The electrophysiological findings in the present study strongly suggest the presynaptic regulation of glutamate and GABA release through H_3_ receptors. Electron microscopy and immunohistochemistry are potent tools for providing direct evidence of the existence of H_3_ receptors in glutamatergic or GABAergic terminals. We performed pre-embedding electron microscopy and immunohistochemistry for the H_3_ receptor and GAD, a GABA-synthesizing enzyme that catalyzes the decarboxylation of glutamate, or for H_3_ receptors and VGLUT1, a suitable marker for immunolabeling glutamate terminals.

**FIGURE 8:**
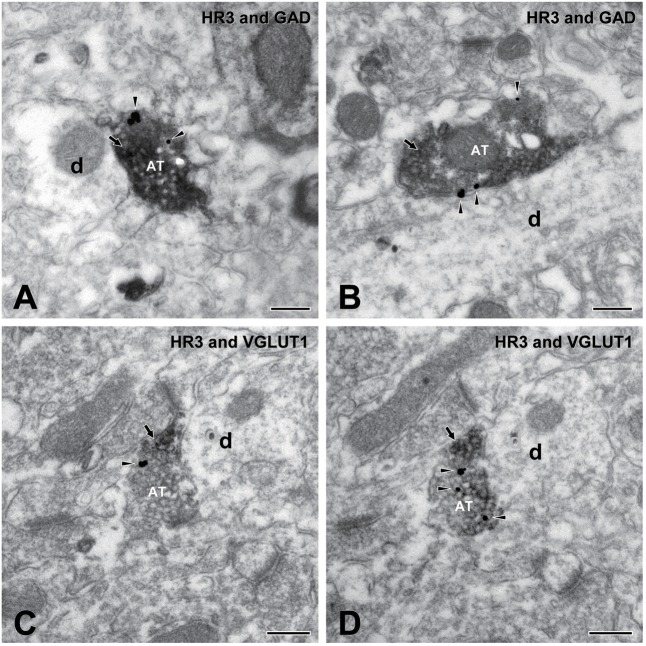
Electron micrographs showing double immunostaining for H_3_ receptors and glutamic acid decarboxylase (GAD) and for H_3_ and vesicular glutamate transporter 1 (VGLUT1). **(A,B)** An axon terminal (AT) presynaptic to dendrite (d) shows immunoreactivity for both H_3_ receptors (silver-gold labeling, arrowheads) and GAD (immunoperoxidase labeling, arrow). **(C,D)** Electron micrographs of an AT in adjacent ultrathin sections: An AT shows immunoreactivity for both H_3_ receptors (silver-gold labeling, arrowheads) and VGLUT1 (immunoperoxidase labeling, arrow). Note the consistency of immunostaining in adjacent ultrathin sections of **(C,D)**. Scale bar = 200 nm.

**Figures 8A,B** show two examples of the observed localization of H_3_ receptors on the GAD-immunopositive terminals in the dysgranular IC. H_3_ receptors and GAD were labeled with silver-gold (arrowheads) and immunoperoxidase (arrows), respectively. Similarly, H_3_ receptors labeled with silver-gold (arrowheads) were occasionally observed on VGLUT1-immunopositive terminals, as shown in **Figures 8C,D**. Most presynaptic GAD/VGLUT1-immunopositive axons with H_3_ receptors terminated to middle-sized dendrites.

### RAMH-Induced Modulation of Excitatory Propagation in the IC

To elucidate the functional role of H_3_ receptors in cortical information processing *in vivo*, we examined the effects of RAMH on excitatory propagation in the IC by the optical imaging technique using a voltage-sensitive dye. As previously we reported, repetitive electrical stimulation of the DI rostral to the MCA, i.e., the gustatory cortex, consistently evokes excitation that propagates rostrocaudally, which is principally mediated by AMPA and GABA_A_ receptors (Fujita et al., 2010, 2011, 2012; Mizoguchi et al., 2011). Therefore, we used the same protocol to evaluate the effects of RAMH on glutamatergic and GABAergic synaptic transmission in the IC.

**FIGURE 9:**
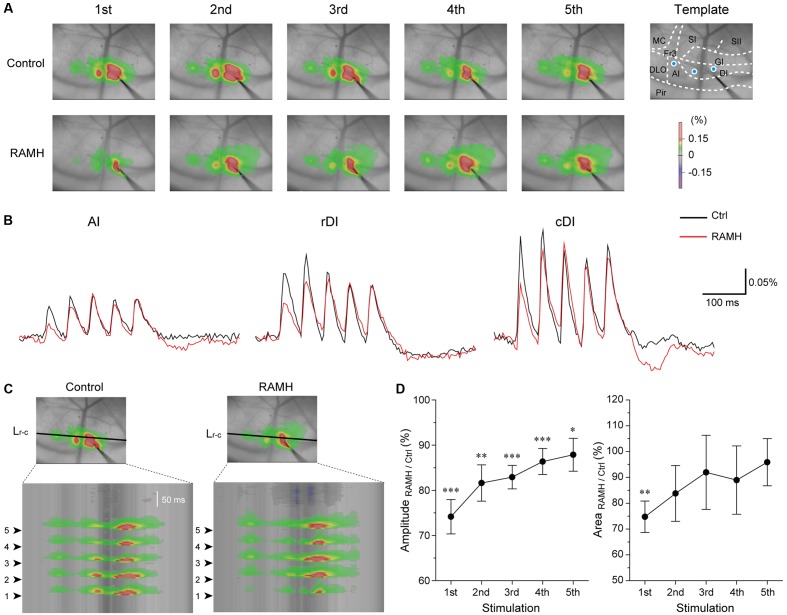
Effects of RAMH on excitatory propagation induced by repetitive stimulation in the IC. **(A)** Color-coded optical signals induced by a train of 5 pulses at 20 Hz in control (upper panels) and during application of RAMH (10 μM). Note that RAMH suppressed optical signal amplitude response to the 1st stimulus but increased those to the 2nd to 5th stimuli proximal to the stimulation site. A template of cortical subdivisions of the IC and surrounding regions is shown in the right panel of control. Blue circles in the AI, the rostral DI (rDI) and the caudal DI (cDI) indicate the locations of regions of interest (ROIs). **(B)** Optical responses in AI, rDI, and cDI in control (black) and during RAMH application (red). The ROIs are shown in the top left panel of **(A)**. **(C)** Spatiotemporal maps along the horizontal black line shown in the top panel at the 3rd in **(A)**. Arrowheads on the left indicate the timing of electrical stimulation. Note that RAMH suppressed optical signals responding to the 1st stimulus. **(D)** Normalized amplitude and area of the effect of RAMH on excitatory propagation responding to the 1st to 5th stimuli. Note that RAMH-induced suppression of amplitude and area was prominent in the 1st response. AI, agranular IC; DI, dysgranular IC; DLO, dorsolateral orbital cortex; Fr3, frontal cortex (area 3); GI, granular IC; MC, motor cortex; Pir, piriform cortex; SI, primary somatosensory cortex; SII, secondary somatosensory cortex. ^∗^*P* < 0.05, ^∗∗^*P* < 0.01, ^∗∗∗^*P* < 0.001, paired *t*-test.

Electrical stimulation of the dysgranular IC (5 train of pulses at 20 Hz, 7 V) evoked excitatory propagation that spread parallel to the rhinal fissure (**Figure 9A**). The amplitude of excitation was largest around the stimulation electrode (Δ*F/F* = 0.25 ± 0.03%, *n* = 12; **Figure 9B**). Application of RAMH (10 μM) to the cortical surface decreased the amplitude between the bottom to peak of optical signals responding to the 1st stimulus (*n* = 12; *P* < 0.001, paired *t*-test; **Figures 9A–C**). Although the RAMH-induced suppression of the amplitude was also observed in the responses to the 2nd to 5th stimuli, the 1st responses showed the most prominent suppression by RAMH and the suppression was gradually recovered in the following responses (**Figure 9D**). In parallel to the RAMH-induced suppression of the excitation amplitude, the area of excitation was decreased in the 1st responses from 11.1 ± 0.8 mm^2^ to 8.3 ± 0.9 mm^2^ (*n* = 11, *P* < 0.01, paired *t*-test), and the latter responses showed less decrease in the excitation area (*P* = 0.06–0.41, paired *t*-test; **Figure 9D**). These results suggest that RAMH suppresses excitatory propagation especially responding to the 1st stimulation, though the RAMH-induced suppression is less in the latter responses.

## Discussion

In the present study, we examined the functional roles of H_3_ receptors in local circuits in the rat IC and investigated how the H_3_ receptor-dependent synaptic modulation regulates the excitatory propagation *in vivo* preparations. An H_3_ receptor agonist suppressed both uEPSCs and uIPSCs, regardless of presynaptic and postsynaptic neuron subtypes, and these effects were likely mediated through H_3_ heteroreceptors in glutamatergic and GABAergic presynaptic terminals. These H_3_ receptor-mediated modulations of synaptic transmission suppress excitatory propagation in the IC, and thus, H_3_ heteroreceptors likely regulate cortico-cortical information processing in coordination with H_3_ autoreceptors.

### H_3_ Receptors in the IC

Histaminergic axons derived from the TMN widely project to the cerebral cortex, including the IC (Haas et al., 2008). Previous autoradiographic studies using the H_3_ receptor agonists, [^3^H]RAMH or N alpha-[^3^H]methylhistamine, have demonstrated the rostrocaudal gradient of H_3_ receptor density (Pollard et al., 1993), and the IC shows the highest abundance of H_3_ receptor expression in the cerebral cortex (Pollard et al., 1993; Cumming et al., 1994). Consistently, the *in situ* hybridization of H_3_ receptor mRNA combined with autoradiographic mapping of the H_3_ receptor antagonist, [^125^I]iodoproxyfan, demonstrated high concentration of H_3_ receptors in the cerebral cortex, particularly in the IC (Pillot et al., 2002). Therefore, the IC is considered as the best target to examine the physiological roles of H_3_ receptors in the cerebral cortex.

In contrast to the subcellular distribution patterns of H_3_ receptors in the TMN, the distinct spatial patterns of H_3_ receptors in cerebrocortical neurons remain unclear. The present study corroborates previous reports of H_3_ receptor expression in the IC and further demonstrates that H_3_ receptors are expressed in VGLU1- or GAD-immunopositive terminals. Several studies have demonstrated the expression of H_3_ heteroreceptors in glutamatergic terminals of the dentate gyrus (Brown and Reymann, 1996; Doreulee et al., 2001) and striatum (Doreulee et al., 2001) or GABAergic terminals in the hypothalamus (Jang et al., 2001). However, these electrophysiological findings only implicate the presynaptic functions of H_3_ receptors. Thus, electron microscopy and immunohistochemistry performed in the present study provides a direct anatomical evidence of H_3_ heteroreceptors in the cerebral cortex.

### H_3_ Heteroreceptor-Mediated Modulation of Synaptic Transmission

An *in vitro* whole-cell patch-clamp study in the rat dentate gyrus demonstrated that histamine or RAMH reduces the amplitude of evoked EPSCs and increases in PPR (Brown and Reymann, 1996) through a reduction in Ca^2+^ influx (Brown and Haas, 1999). This finding is applicable *in vivo*, as RAMH decreases evoked EPSP amplitude, which is blocked through thioperamide, in the dentate gyrus (Manahan-Vaughan et al., 1998). Similarly, the H_3_ heteroreceptor-mediated suppression of evoked EPSPs/EPSCs has been reported in the striatum (Doreulee et al., 2001) and basolateral amygdala (Jiang et al., 2005). Despite abundant H_3_ receptor expression in the cerebral cortex, there is little evidence showing the functional roles of H_3_ receptors. Microdialysis in the prefrontal cortex demonstrated that thioperamide does not affect the basal release of glutamate and GABA (Welty and Shoblock, 2009). However, GABA release through strong stimulation, such as high potassium or NMDA application, is suppressed with thioperamide (Dai et al., 2006; Welty and Shoblock, 2009).

There are at least three subtypes of GABAergic interneurons in the IC (Kobayashi et al., 2010; Koyanagi et al., 2010; Yamamoto et al., 2010), and the kinetics of postsynaptic currents are different among interneuron subtypes, e.g., FS cells induce much larger uIPSCs in amplitude than LTS cells (Xiang et al., 2002; Kobayashi et al., 2008; Koyanagi et al., 2010). Therefore, the identification of presynaptic and postsynaptic neuron subtypes provides valuable information regarding EPSCs/uIPSC recordings. We consider that paired whole-cell recording has a large advantage in the precise analysis of glutamate/GABA-mediated synaptic transmission, as presynaptic and postsynaptic neuron subtypes can be identified. The present study demonstrated that the H_3_ receptor agonist consistently suppressed the amplitude of uEPSCs/uIPSCs, regardless of presynaptic or postsynaptic neuron subtypes, suggesting that H_3_ receptors are widely distributed in the IC, rather than expressed in specific neurons.

### Intracellular Cascade of Synaptic Modulation through H_3_ Receptors

H_3_ receptors are metabotropic receptors coupled to G_i/o_ proteins (Clark and Hill, 1996). H_3_ receptors reduce intracellular cyclic AMP (cAMP) concentrations through the suppression of adenylyl cyclase (Torrent et al., 2005; Moreno-Delgado et al., 2006). The reduction in the cAMP concentration decreases Ca^2+^ currents via L, N, and P/Q channels (Takeshita et al., 1998), and in turn, H_3_ autoreceptor activation reduces histamine release from presynaptic terminals. Therefore, it is reasonable to speculate that similar mechanisms underlie the regulation of glutamate or GABA release through H_3_ heteroreceptors in the cerebral cortex.

The findings of RAMH-induced suppression of uEPSCs/uIPSCs imply that adenylyl cyclase, in glutamatergic or GABAergic terminals, is constantly activated to maintain intracellular cAMP concentrations, thereby contributing to the facilitatory effects on neurotransmitter release from these terminals. Although several studies supported the findings that H_3_ receptor activation suppresses spontaneous GABA release by inhibiting P/Q channels in the hypothalamus (Jang et al., 2001) and striatum (Jang et al., 2001; Arias-Montano et al., 2007), they suggest an independent mechanism of the adenylyl-cAMP pathway. Further studies are necessary to clarify the second messenger systems involved in H_3_ receptor activation.

The effects of thioperamide on synaptic transmission are controversial. As described above, thioperamide is considered a typical H_3_ receptor antagonist (Arrang et al., 1987), whereas several studies have reported thioperamide as an inverse agonist (Morisset et al., 2000). The present study demonstrates that the application of thioperamide slightly enhanced the uIPSC amplitude.

There are at least two possibilities that explain the thioperamide-induced enhancement of uIPSCs. If histamine is spontaneously released and H_3_ receptors are consistently activated in the slice preparations, then the application of thioperamide might suppress the H_3_ receptor-dependent decrease in the uEPSC/uIPSC amplitude. Second, thioperamide behaves as an inverse agonist. Little effect of JNJ5207852 on uEPSCs and uIPSCs suggests that the 1st possibility is unlikely. The contradictory effects of thioperamide on uEPSCs and uIPSCs also supports the second possibility. Furthermore, in a previous study of IC pyramidal cells, we showed that histamine increases spike firing via H_2_ receptors and RAMH does not change the frequency, suggesting that spontaneous histamine release is less likely in the IC slice preparations (Takei et al., 2012). Therefore, the facilitative effects of thioperamide on the amplitude of uEPSCs/uIPSCs might indicate a role for this molecule as an inverse agonist.

### Functional Significance of H_3_ Receptors *in Vivo*

H_3_ receptors are involved in higher brain functions, such as cognition, learning and memory, sleep, appetite, and energy metabolism, and the disruption of H_3_ receptors induces related neurological disorders (Haas et al., 2008). Based on this evidence, H_3_ receptors have recently been considered as promising targets for the treatment of several neuronal disorders, including hypersomnia (Leurs et al., 2005), eating disorders (Leurs et al., 2005; Steffen et al., 2006), schizophrenia (Ito, 2009), and pain (Cannon et al., 2007). It is interesting to focus on the study that the activation of H_3_ receptors in the IC impairs aversive taste memory formation (Purón-Sierra et al., 2010), because the functional roles of the IC have been implicated in gustatory, visceral, and nociceptive information processing (Yamamoto et al., 1981; Yasui et al., 1991; Ogawa and Wang, 2002; Jasmin et al., 2003; Kobayashi, 2011). Our present findings that activation of H_3_ suppresses glutamate and GABA release from presynaptic terminals may be involved in this process. In addition, recent studies have indicated additional functions for the IC in attention, reasoning, planning, and decision-making processes associated with smoking and drug abuse (Naqvi et al., 2007; Naqvi and Bechara, 2009). Thus, H_3_ antagonists are potential candidates for the improvement of the physiological functions of the IC.

## Author Contributions

MK designed this study. HT, KY, and Y-CB contributed to the acquisition, analysis, and interpretation of the data in this study. TS contributed to the interpretation of the data. KY and MK drafted and wrote the manuscript. All authors have approved the final version of the manuscript. All authors agree to be accountable for all aspects of the work in ensuring that questions related to the accuracy or integrity of any part of the work are appropriately investigated and resolved. All persons designated as authors qualify for authorship, and all those who qualify for authorship are listed.

## Conflict of Interest Statement

The authors declare that the research was conducted in the absence of any commercial or financial relationships that could be construed as a potential conflict of interest.
